# Letter from the Editor in Chief

**DOI:** 10.19102/icrm.2022.130808

**Published:** 2022-08-15

**Authors:** Moussa Mansour


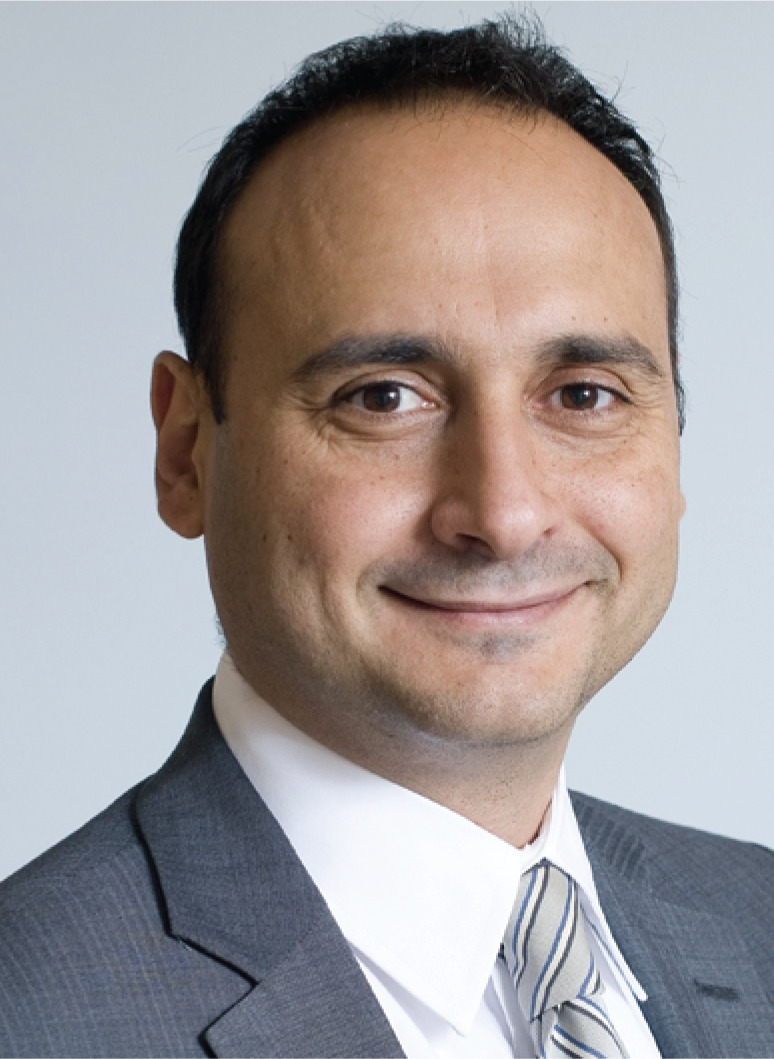


Dear readers,

This issue of *The Journal of Innovations in Cardiac Rhythm Management* contains many interesting original research articles. In particular, I would like to highlight the article by Pothineni et al. titled “Complications of Cardiac Resynchronization Therapy: Comparison of Safety Outcomes from Real-world Studies and Clinical Trials.”^1^ In it, the authors compared the complication rates of cardiac resynchronization therapy (CRT) between 4,442 patients enrolled in randomized controlled trials (RCTs) and 72,554 patients enrolled in “real-world” studies, including registries and administrative databases. They found that the rates of procedural complications with CRT were significantly higher in RCTs compared to the real world (8.1% vs. 6.9%, *P* = .002) and advocated for the implementation of better data-collection methods to accurately capture complications in registries to ensure accurate and reliable public reporting of complications and outcomes.

The findings in this manuscript are not surprising. However, they represent an important reminder about differences in the rigor of data derived from RCTs and those obtained from registries and administrative data collections. Not only do RCTs have far superior statistical methodologies, including randomized prospective designs and sound power calculations; they also have strict follow-up schedules and adverse event reporting. In addition, RCTs are often supervised by data safety and monitoring boards with numerous responsibilities, such as ensuring the integrity of the data and timely reporting. In contrast, registries and databases are largely retrospective collections of data that often rely on voluntary reporting of adverse events.

Designing, conducting, and completing an RCT can be a long and expensive process. As a result, most RCTs in the field of cardiac electrophysiology in recent years have been limited to medication or device studies funded by industry. Although these studies have been critically important for advancing the field, they do not answer many physiology-based treatment questions that do not require the use of novel medical devices or medications. As such, for the benefit of the field of cardiac electrophysiology at large, it is of the utmost importance for professional societies to seek out financial support for RCTs that also address these areas.

Best regards, and I hope that you enjoy reading this issue of the journal.


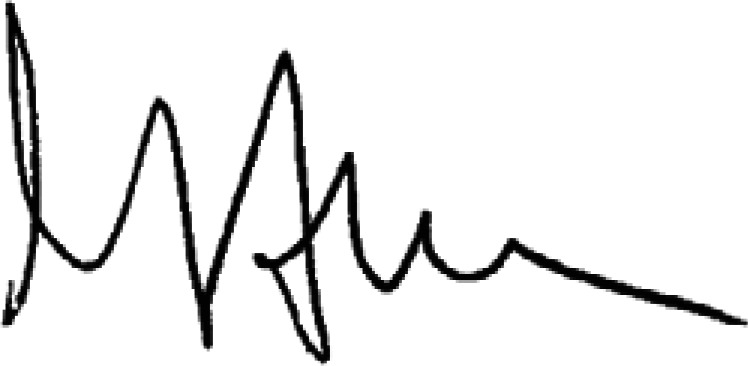


Sincerely,

Moussa Mansour, md, fhrs, facc

Editor in Chief

*The Journal of Innovations in Cardiac Rhythm Management*

MMansour@InnovationsInCRM.com

Director, Atrial Fibrillation Program

Jeremy Ruskin and Dan Starks Endowed Chair in Cardiology

Massachusetts General Hospital

Boston, MA 02114
